# Real-world experience with INTERLACE-style induction chemotherapy: Tumor regression and MRI-guided brachytherapy in locally advanced cervical cancer

**DOI:** 10.1016/j.gore.2026.102231

**Published:** 2026-10-13

**Authors:** Ashley Chavana, Madeline Flanagan, Amir Owrangi, Shelly Sharma, Claire Hoppenot, Jan Sunde, Anthony B. Costales, Spoorthi Kamepalli, Tracilyn Hall, Michelle Ludwig

**Affiliations:** a Department of Radiation Oncology, Dan L Duncan Comprehensive Cancer Center, Baylor College of Medicine, 1919 Old Spanish Trl, Houston, TX, 77054, USA; b Department of Gynecologic Oncology, Baylor College of Medicine, One Baylor Plaza, Houston, TX, 77030, USA; c Department of Obstetrics and Gynecology, Duke University Medical Center, 10 Duke Medicine Circ Durham, NC, 27710, USA; d Department of Radiation Oncology, University of Colorado Anschutz Medical Campus, 1665 Aurora Court, Aurora, CO, 80045, USA

**Keywords:** Cervical cancer, Brachytherapy, Induction chemotherapy, MRI-guided adaptive brachytherapy, Real-world outcomes

## Abstract

**Objective:**

Achieving optimal brachytherapy coverage for bulky cervical tumors (>30 cc) remains challenging, and the ideal treatment approach is unclear. This study describes real-world tumor volume response, brachytherapy dose delivery, and toxicity in patients treated with an INTERLACE-style induction chemotherapy regimen followed by chemoradiation and MRI-guided adaptive brachytherapy.

**Methods:**

This retrospective, two-institution cohort included eight patients with locally advanced cervical cancer treated with weekly carboplatin/paclitaxel induction chemotherapy followed by cisplatin-based chemoradiation and MRI-guided brachytherapy. All three serial tumor measurements (at diagnosis, after induction chemotherapy, and after chemoradiation/before brachytherapy) were obtained by pelvic MRI. Gross tumor volumes (GTV) were contoured by the treating radiation oncologist; organ-at-risk volumes were delineated by treating physicians.

**Results:**

FIGO stage was IIIC1 in 6 patients, IIA in 1, and IB3 in 1. Five patients (62.5%) were PD-L1 positive. Seven patients received tandem-and-ovoid brachytherapy and one received a Syed-Neblett interstitial implant. Median GTV was 88.3 cc (range 18–370) at diagnosis, 15.3 cc (range 0–187.1) after induction chemotherapy, and 2.2 cc (range 0–11.5) after chemoradiation. Median per-patient GTV reduction was 70% (range, 45–100%) after induction chemotherapy alone and 97% (range 74–100%) by completion of chemoradiation. Seven patients presented with bulky disease (>30 cc); three remained bulky after induction chemotherapy and none after chemoradiation. Median high-risk clinical target volume (HR-CTV) D90 cumulative dose in 2-Gy fraction equivalents (EQ.D2) was 90.5 Gy (range 87–93.7) and median HR-CTV D98 was 79.8 Gy EQ.D2 (range 77.2–84.8; *n* = 7). Grade ≥ 3 hematologic toxicity was more common during chemoradiation than during induction chemotherapy (neutropenia in 3/8 [37.5%] vs 1/8 [12.5%]; leukopenia in 2/8 [25%] vs 0/8 [0%]). Thrombocytopenia (1/8,12.5%) and one case of anemia were observed exclusively during induction chemotherapy. Two patients discontinued cisplatin early because of hematologic toxicity. Seven of eight patients achieved a complete metabolic response on PET at 3 months post-treatment and remained disease-free at last follow-up (median 62.4 months; range 23.0–72.9).

**Conclusion:**

In this real-world cohort, INTERLACE-style induction chemotherapy produced substantial tumor regression that facilitated tandem-and-ovoid brachytherapy in most patients while achieving EMBRACE-aligned HR-CTV doses with manageable, phase-specific toxicity. These hypothesis-generating findings warrant confirmation in larger, prospective, and comparative cohorts.

## Highlights

• ICT reduced tumor volume in bulky cervical cancer.
• Median GTV reduction was 70% after ICT and 97% after CRT.
• MRI-guided brachytherapy with an EQ.D2 ~ 90 Gy was achieved with minimal toxicity.
• INTERLACE regimen combined with EMBRACE-style brachytherapy is feasible.
• This work supports dose escalation for bulky tumors using an MRI-guided approach.

## 1 Introduction

Cervical cancer is the third most common gynecologic malignancy in the United States, with approximately 14,000 new cases diagnosed annually (American Cancer Society, n.d.-a). The incidence is disproportionately higher among Hispanic and African American women, likely reflecting disparities in access to human papillomavirus vaccination and preventive care; these disparities are compounded by geographically uneven access to brachytherapy itself, including within Texas (Jhavar et al., 2026). While the overall five-year survival rate across all stages is approximately 68% (American Cancer Society, n.d.-b), outcomes are substantially worse for patients with locally advanced disease (FIGO stage IB3–IVA). In a study by Wright et al., five-year survival was 76.1% for patients with stage IB3 disease but declined steadily with advancing stage, reaching 24% for stage IVA (Wright et al., 2019). Because distant failure remains a challenge in locally advanced cervical cancer, systemic treatment intensification has been an ongoing focus of investigation (Hathout et al., 2025).

Concurrent chemoradiation followed by brachytherapy remains the standard curative approach for locally advanced cervical cancer, and outcomes are closely tied to the quality of brachytherapy delivery. Contemporary MRI-guided adaptive brachytherapy supports individualized target delineation and dose optimization and has demonstrated durable local control with acceptable morbidity in a prospective multicenter experience (Pötter et al., 2021). However, achieving optimal brachytherapy coverage can be challenging in patients presenting with bulky tumors, where unfavorable geometry and proximity of organs at risk may limit target coverage without exceeding normal tissue constraints. Consistent with EMBRACE-aligned principles, volumetric planning aims for a high-risk clinical target volume (HR-CTV) equivalent dose in 2 Gy fractions of 90–95 Gy, with lower acceptable targets in selected scenarios such as smaller residual disease after chemoradiation (Pötter et al., 2018).

Emerging randomised evidence suggests that induction chemotherapy before chemoradiation may improve both progression-free and overall survival. In the INTERLACE trial, induction chemotherapy prior to chemoradiation improved outcomes compared with chemoradiation alone in patients with locally advanced cervical cancer (McCormack et al., 2024). Notably, prior intensification strategies using adjuvant chemotherapy after chemoradiation did not demonstrate a survival benefit, emphasizing the importance of treatment sequencing (Mileshkin et al., 2023). More recently, immunotherapy combined with chemoradiation has also improved outcomes in high-risk locally advanced cervical cancer, further highlighting a rapidly evolving treatment landscape and increasing interest in multimodality regimens (Lorusso et al., 2024). An ongoing cooperative-group trial (NRG-GY037) is further evaluating induction chemotherapy in this setting, underscoring the timeliness of real-world data on tumor response, brachytherapy technique, and toxicity with this approach.

Building on these data, we sought to evaluate outcomes of an INTERLACE-style induction chemotherapy regimen followed by chemoradiation and MRI-guided brachytherapy delivered according to EMBRACE principles in patients treated at two institutions in Houston, Texas. We focused on tumor volume response across treatment, delivered brachytherapy dose, and treatment-related toxicities.

## 2 Methods

This retrospective, multi-institutional cohort study included review of patients with locally advanced cervical cancer treated between November 2023 and August 2024 at the Harris Health System and the Dan L. Duncan Comprehensive Cancer Center. The study protocol was approved by the institutional review boards of both institutions (H-47373). Informed consent was waived due to the retrospective study design.

Demographic (age, ethnicity, insurance status), clinical (diagnosis, FIGO stage, histology, PD-L1 status), and treatment-related data were abstracted by trained personnel at each site. Adverse events were graded according to the Common Terminology Criteria for Adverse Events, version 5.

All patients received induction chemotherapy with weekly carboplatin (area under the receiver operator curve of 2) and paclitaxel (80 mg/m^2^) for 6 weeks followed by cisplatin-based chemoradiation and MRI-guided brachytherapy. Radiation treatment began with pelvic ± para-aortic external beam radiotherapy using a conventional fractionation schedule of 45 Gy delivered in 25 fractions over 5 weeks. Grossly involved lymph nodes received a simultaneous integrated boost to 55 Gy for true pelvic nodes or 57.5 Gy for nodes outside of the true pelvis, consistent with EMBRACE II protocol. After completing external beam radiation, brachytherapy was initiated.

During the final week of external beam radiotherapy, a pelvic MRI was obtained with T2-weighted sagittal, axial, and coronal sequences. Before brachytherapy applicator insertion, a bladder catheter was placed and left open during image acquisition and treatment delivery to ensure consistent bladder emptying and reproducibility. Following applicator insertion, a CT scan was performed to guide catheter reconstruction. The CT images were registered with the previously acquired MRIs (obtained prior to induction chemotherapy and prior to chemoradiation) to enable accurate delineation of the gross tumor volume, high-risk clinical target volume, and, when indicated, intermediate-risk clinical target volume. Organs at risk including the bladder, rectum, sigmoid colon, and bowel were also contoured by the treating radiation oncologist.

Treatment planning was performed using a high-dose-rate Ir-192 source with the TG-43 formalism in the BrachyVision v18 planning system (Varian Medical Systems, Palo Alto, CA). Plans were optimized to provide adequate dose coverage to the high-risk and intermediate-risk clinical target volumes while respecting tolerance limits for adjacent organs at risk. Previous external beam doses were incorporated and converted to equivalent doses in 2 Gy fractions using the linear-quadratic model (α/β = 10 Gy for tumor and α/β = 3 Gy for organs at risk) for each patient. Our institution follows EMBRACE-II protocol for organ-at-risk constraints and target coverage. Specifically, we aim for the cumulative dose to 90% volume (D90) of the high-risk clinical target volume (HR-CTV) to exceed 85–90 Gy equivalent dose in 2-Gy fractions (EQ.D2) when combining external beam radiation and brachytherapy.

Gross tumor volumes (at diagnosis, after induction chemotherapy, and after chemoradiation) were contoured using CT simulation with fused MRI and measured by the treating radiation oncologist; these serial measurements track the visible/measurable tumor (GTV) rather than the HR-CTV. The HR-CTV, which is the target for EMBRACE-style dose reporting, is a broader volume that by definition includes the whole cervix and any residual pathologic parametrial or adjacent tissue, not only the shrinking gross tumor. We therefore report GTV regression as our volumetric endpoint and report HR-CTV D90/D98 separately as our dosimetric endpoint; HR-CTV volume itself (in cc) at the time of brachytherapy was not systematically retained for this cohort and is not reported. Total change in GTV was obtained by subtracting the post-chemoradiation tumor volume from the pre-induction chemotherapy volume.

## 3 Results

Eight patients with locally advanced cervical cancer were included (Table 1), all of whom were treated between November 2023 and August 2024. Eight additional patients with cervical cancer who were treated with neoadjuvant chemotherapy followed by chemoradiation between September 2024 and February 2026 were identified; however these patients were excluded due to extensively advanced disease (FIGO stage IVA-IVB), ineligibility for brachytherapy due to extensive bladder involvement or distal disease, or induction chemotherapy regimen differing from INTERLACE-style induction.

**Table 1:** Patient, Tumor, and Treatment characteristics.

Patient, Tumor, & Treatment Characteristics (*n* = 8)	Value
Age, median (range)	55 (32–66)
FIGO stage, n (%)	
IIIC1	6 (75.0%)
IIA	1 (12.5%)
IB3	1 (12.5%)
Ethnicity, n (%)	
Hispanic	4 (50%)
Mexican/Chicano	1 (12.5%)
African American	1 (12.5%)
Caucasian	1 (12.5%)
Other	1 (12.5%)
HPV status/type, n (%)	
HPV-positive disease, p16+	4 (50%)
HPV-positive with unknown p16/p18 status	2 (25%)
HPV-negative disease	2 (25%)
Histologic grade, n (%)	
Squamous cell carcinoma	6 (75%)
Adenocarcinoma	2 (25%)
Nodal status	
Gross node(s) present before & after induction	6 (75%)
Node-negative	2 (25%)
PD-L1 status, n (%)	
Positive	5 (62.5%)
Negative	1 (12.5%)
Unknown	2 (25.0%)
Brachytherapy applicator, n (%)	
Tandem & ovoid	7 (87.5%)
Syed-Neblett interstitial	1 (12.5%)

The median age at diagnosis of the 8 included patients was 55 years. Half of the cohort (50%) was Hispanic. FIGO stage was IIIC1 in 6 patients (75%), IIA in 1 (12.5%), and IB3 in 1 (12.5%). PD-L1 status was assessed via immunohistochemistry in 75% and was positive in 5, negative in 1, and unknown in 2 patients. No patient received pembrolizumab or other immunotherapy prior to or during the treatment course. Six patients' histology was squamous cell carcinoma with 4 of 6 with HPV-associated squamous cell carcinoma and 2 of 6 with HPV-negative squamous cell carcinoma. Two patients' histology was adenocarcinoma with cervical gastric type adenocarcinoma in one and HPV associated adenocarcinoma in the other. In the five patients with HPV-positive disease, p16 was positive in 3 and in the other 2 patients p16/p18 status was unknown.

Six of the eight patients had node positive disease, and all six of these patients received simultaneous integrated boost of 55 Gy in 25 fractions to gross pelvic lymph nodes. All 8 patients were initially planned for likely or possible interstitial brachytherapy following chemoradiation, and seven ultimately underwent brachytherapy with a tandem-and-ovoid applicator; one patient with the largest baseline tumor (370 cc) required a Syed-Neblett interstitial implant (Table 1).

The mean overall treatment time was 47 days (median 47; range 41–56), with a median interval of 7.5 days (range 0–75) from the completion of induction chemotherapy to the initiation of chemoradiation (Table 2). One patient (patient 6) required 56 days to complete treatment due to machine downtime necessitating the rescheduling of a brachytherapy session. Six patients missed at least one radiotherapy fraction (range: 1–4 sessions), though none of these interruptions were attributable to treatment-related toxicities. Missed sessions were primarily due to quality assurance issues, incomplete treatment planning, insurance delays, or weather-related disruptions.).

**Table 2:** Radiation treatment details, including HR-CTV D90 and D98 (equivalent dose in 2 Gy fractions).

Patient #	Time to start of RT (days)	RT duration (days)	Missed RT appt.	HR-CTV D90 (Gy)	HR-CTV D98 (Gy)
1	8	48	No	88.9	79.0
2	33	49	Yes	90.9	82.0
3	26	41	Yes	87.0	78.0
4	7	46	Yes	90.8	82.4
5	0	44	Yes	88.0	77.2
6	75	56	Yes	90.3	79.8
7	7	48	Yes	93.7	84.8
8	6	45	No	92.4	n/a
Median (range)	7.5 (0–75)	47 (41–56)	–	90.5 (87–93.7)	79.8 (77.2–84.8)

Abbreviations: RT, radiotherapy; HR-CTV, high-risk clinical target volume

For the HR-CTV, the mean dose to 98% of the volume (D98) was 80.5 Gy EQ.D2 (median 79.8; range, 77.2–84.8), and the mean D90 was 90.2 Gy EQ.D2 (median 90.5; range 87–93.7). In contrast, the mean D98 for the intermediate risk clinical target volume (IR-CTV) based on pre-induction chemotherapy volume was 59 Gy (median 59.9;range, 55.2–61.8 Gy) (Table 2).

Three patients completed ≥5 cycles of concurrent cisplatin, and four completed exactly 4 cycles. Two patients (patients 2 and 6) discontinued cisplatin prematurely due to hematologic adverse events. Grade 3 neutropenia occurred in 1/8 patients during induction versus 3/8 during chemoradiation, grade 3 leukopenia in 0/8 versus 2/8, and grade 3 thrombocytopenia occurred in 1/8 versus 0/8, respectively (Table 3a, Table 3b). One patient experienced grade 3 anemia which occurred only during induction chemotherapy, while another patient experienced grade 3 anemia during chemoradiation (1/8 versus 1/8) (Table 3a, Table 3b). Supportive interventions included granulocyte colony-stimulating factor for one patient during chemoradiation, and red blood cell transfusion was required in one patient during both induction chemotherapy and chemoradiation(Table 3a, Table 3b).

**Table 3a:** Hematologic toxicity during induction chemotherapy, graded per Common Terminology Criteria for Adverse Events, Version 5 (CTCAE v5) standards.

Patient #	Neutropenia	Leukopenia	Thrombocytopenia	Anemia	GCSF given	Transfusion given
1	no	no	no	no	no	no
2	no	no	no	no	no	no
3	no	no	no	no	no	no
4	no	no	no	no	no	no
5	no	no	no	no	no	no
6	grade 3	no	grade 3	no	no	no
7	no	no	no	no	no	no
8	no	no	no	grade 3	no	yes
Grade ≥ 3, n (%)	1 (12.5%)	0 (0.0%)	1 (12.5%)	1 (12.5%)	0/8	1/8

**Table 3b:** Hematologic toxicity during concurrent chemoradiation, graded per Common Terminology Criteria for Adverse Events, Version 5 (CTCAE v5) standards.

Patient #	Neutropenia	Leukopenia	Thrombocytopenia	Anemia	GCSF given	Transfusion given
1	no	no	no	no	no	no
2	no	no	no	no	no	no
3	grade 3	grade 3	no	no	yes	no
4	no	no	no	no	no	no
5	no	no	no	no	no	no
6	grade 3	grade 3	no	no	no	no
7	no	no	no	no	no	no
8	grade 3	no	no	grade 3	no	yes
Grade ≥ 3, n (%)	3 (37.5%)	2 (25.0%)	0 (0.0%)	1 (12.5%)	1/8	1/8

The mean gross tumor volume was 122 cc at diagnosis (median 88.3 cc, range 18–370), 43 cc after induction chemotherapy (median 15.3 cc, range 0–187.1), and 3 cc after chemoradiation (median 2.2, range 0–11.5). Median per-patient GTV reduction was 70% (range 45–100%) after induction chemotherapy and 97% (range 74–100%) after chemoradiation. Seven patients presented with bulky disease (>30 cc) at baseline; this decreased to three patients after induction chemotherapy, and none following chemoradiation (Table 4, example shown in Fig. 1).

**Table 4:** Gross tumor volumes (GTV) before induction chemotherapy (ICT), after ICT, and after chemoradiation (CRT), with per-patient percent reduction.

Patient #	Pre-ICT vol. (cc)	Post-ICT vol. (cc)	Post-CRT vol. (cc)	% reduction after ICT	% reduction after CRT (cumulative)
1	18	7	4.7	61%	74%
2	99.9	0	0	100%	100%
3	69.5	13.2	2.4	81%	97%
4	157.6	61.6	2	61%	99%
5	370	187.1	11.5	49%	97%
6	82.5	5.3	5.2	94%	94%
7	92.5	50.8	1.8	45%	98%
8	84.2	17.4	0	79%	100%
Median (range)	88.3 (18–370)	15.3 (0–187.1)	2.2 (0–11.5)	70% (45–100%)	97% (74–100%)

**Fig. 1:**
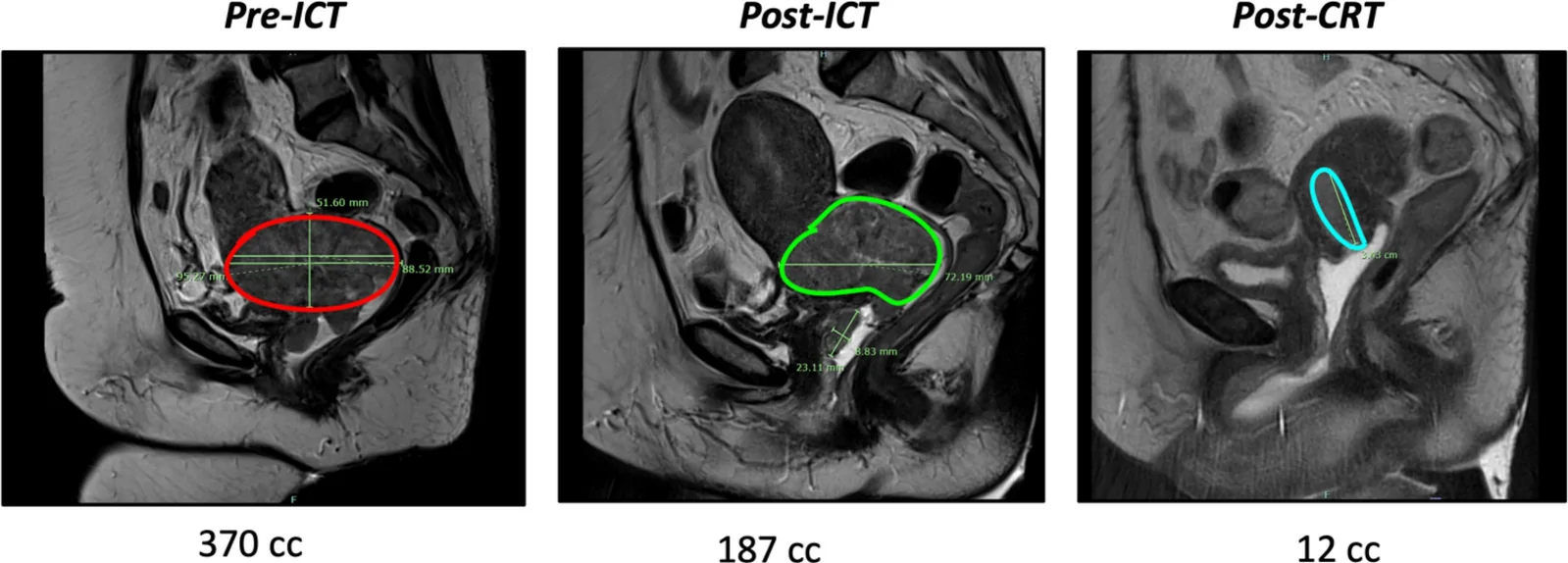
Example images of pre-induction chemotherapy, post-induction chemotherapy, and post-chemoradiation tumor volumes on MRI (patient 5).

Mean maximum dose to 2 cc volume (D2cc) for organs at risk were 61.8 Gy for rectum (median 62.5, range 51.4–69.5), 78.2 Gy for bladder (median 77.8, range 67.1–87.1), 60.7 Gy for bowel (median 60.9 range 48.6–71.3), and 64.8 Gy for sigmoid colon (median 64.5, range 59.5–72.5) (Table 5).

**Table 5:** D2cc for organs at risk (OARs).

Patient #	Rectum D2cc	Bladder D2cc	Bowel D2cc	Sigmoid D2cc
1	69.5	81.1	48.6	72.5
2	65.1	84.9	52.7	62.1
3	62.8	87.1	55.8	67
4	61.1	74.4	68.8	68.7
5	67.3	67.1	71.3	59.5
6	62.2	87.1	66.6	67.8
7	54.7	73.5	62.2	60
8	51.4	70.1	59.5	60.9
Median (range)	62.5 (51.4–69.5)	77.8 (67.1–87.1)	60.9 (48.6–71.3)	64.5 (59.5–72.5)

Seven patients achieved a complete metabolic response on PET imaging at 3 months post-treatment. At last follow-up (mean 53.9 months; median 62.4; range, 23.0–72.9), all seven remained without evidence of disease. One of the eight total patients was lost to follow-up after treatment completion. While the patient's MRI prior to brachytherapy showed a near complete response, a 3-month PET scan was not performed because the patient was lost to follow-up. She re-presented symptomatically 11 months later with biopsy-proven persistent versus recurrent disease and received 6 cycles of quadruplet therapy with carboplatin, paclitaxel, bevacizumab, and pembrolizumab, with near-complete response on subsequent imaging; she is currently receiving maintenance therapy with bevacizumab/pembrolizumab.

## 4 Discussion

In this retrospective, two-institution cohort analysis, we evaluated eight patients with bulky, locally advanced cervical cancer treated with an INTERLACE-style induction chemotherapy regimen followed by EMBRACE-style MRI-guided brachytherapy. GTV regression was substantial and progressive across treatment, and seven of eight patients achieved a complete metabolic response at 3 months and remained without evidence of disease at last follow-up with median follow up over 5 years. One patient was lost to follow-up and re-presented with persistent versus recurrent disease, subsequently with disease controlled with quadruplet salvage therapy and immunotherapy. These findings describe the feasibility and tolerability of this combined approach in a real-world, safety-net setting; given the small cohort, they should be interpreted as hypothesis-generating rather than as evidence of efficacy.

Compared to the INTERLACE trial, our cohort had a similar interval between the end of induction chemotherapy and the start of chemoradiation (median 7.5 days vs 7 days in INTERLACE) and average overall treatment duration (47 vs 45 days, respectively). Notably, we delivered a higher cumulative HR-CTV EQ.D2 from external beam radiation and brachytherapy (87–93.7 vs 78–86) consistent with EMBRACE-aligned targets, while maintaining manageable toxicity. Treatment completion rates were comparable, with 87.5% of patients completing all six cycles of carboplatin and paclitaxel (84% in INTERLACE); one patient discontinued after four cycles due to thrombocytopenia. Similarly, 87.5% of patients received at least four cycles of concurrent cisplatin (85% in INTERLACE), yet the proportion experiencing grade 3 or higher adverse events was lower in our series (37.5% vs 59%).

### 4.1 Contextualizing findings with a historical cohort

Because there ispublished literature on patients with locally advanced cervical cancer treated at our institution who did not receive induction chemotherapy, we can contextualize our findings against these sameinstitution and regional cohorts. Hanania et al. reported a single-implant, inversely and adaptively planned Syed-Neblett interstitial brachytherapy series at our institution (*n* = 40, 25 primary cervical cancers, treated 2014–2017, predating the practice of induction chemotherapy) with a median HR-CTV EQ.D2 D90 of 92 Gy and 2-year overall survival, local control, and progression-free survival of 81%, 81%, and 64%, respectively (Hanania et al., 2019). In that pre-induction-chemotherapy era, essentially all bulky/advanced cases requiring a brachytherapy boost were treated with an interstitial approach.By contrast, only 1 of 8 patients (12.5%) in our INTERLACE-style cohort ultimately required an interstitial (Syed-Neblett) implant, with the remainder converting to tandem-and-ovoid brachytherapy after substantial tumor regression. This isconsistent with the hypothesis that induction chemotherapy may reduce reliance on interstitial technique in appropriately selected patients.

Dong et al. similarly reported a sameinstitution cohort of 45 patients with bulky (HR-CTV >30 cc) cervical cancer undergoing interstitial brachytherapy without induction chemotherapy, of whom 62% achieved a 3-month complete metabolic response (Dong et al., 2024). Our cohort's 87.5% (7/8) 3-month complete metabolic response rate, despite comparably bulky baseline disease (7/8 patients >30 cc), is numerically higher, though the small sample size and differing brachytherapy technique preclude a firm comparison. Shi et al. reported hematologic toxicity in a sameinstitution cohort of 121 stage I–III patients treated with definitive chemoradiation without induction chemotherapy (grade ≥ 3 leukopenia 17%, anemia 9%) (Shi et al., 2022). Our cohort's toxicity (grade ≥ 3 leukopenia 25% [2/8], anemia 25% [2/8]) trended numerically higher, which merits attention to the potential for meaningfully increased toxicity with INTERLACE-style induction chemotherapy; however, our very small sample limits interpretation and did not translate into excess treatment discontinuation. Hsieh et al. described a 119patient safety-net cohort (FIGO IB2-IVA) treated with definitive chemoradiation, with a mean treatment duration of 56.5 days and a 94.1% radiotherapy completion rate (Hsieh et al., 2015). Our cohort's shorter mean overall treatment time (47 days) suggests that incorporating induction chemotherapy and contemporary MRI-guided brachytherapy did not prolong total treatment duration relative to this historical safety-net benchmark. Finally, in Yoder et al.'s prospective quality-of-life and adherence prospective cohort at the same safety-net system (2017–2021, *n* = 93) 15% of patients were FIGO stage I, 46% were stage II, 3% were stage III, and 34% of patients had interstitial brachytherapy, demonstrating how in the absence of induction chemotherapy, a substantial proportion of patients required interstitial brachytherapy (Yoder et al., 2023).

Induction chemotherapy may meaningfully reduce tumor volume to allow brachytherapy with tandem and ovoid rather than interstitial which can spare patients a more invasive, potentially complex procedure. At our institution, intracavitary brachytherapy is performed in clinic, while interstitial brachytherapy requires a placement procedure under general anesthesia at an off-site hospital operating room, followed by admission to the hospital, and multiple ambulance transportations over 2–3 days for simulations, planning, and treatment delivery, all while the patient remains immobilized with perineal interstitial needles in place. Therefore, interstitial brachytherapy has the added risks from general anesthesia, potential infection from needle/catheter placement, thrombosis from prolonged immobilization, and bleeding from device removal.

However, residual disease plus uninvolved cervix is included in the HR-CTV while all initial disease is included in the intermediate risk CTV (IR-CTV) per EMBRACE protocol, so induction therapy does not generally permit dose deescalation to the cervix. There is currently no validated strategy for selectively de-escalating brachytherapy dose based on chemotherapy-related GTV response; however, the EMBRACE II protocol itself incorporates response- and volume-adapted principles for brachytherapy.

### 4.2 Strengths and weaknesses

A key strength of our study is the use of serial MRI tumor volumetrics at three clinically relevant timepoints demonstrating substantial tumor regression over the treatment course, together with granular, phase-specific dosimetric and toxicity data. The cohort also achieved HR-CTV dose delivery aligned with EMBRACE-style targets while maintaining acceptable organ-at-risk doses, manageable toxicity, and lack of added delays. Treatment was delivered across two institutions, improving generalizability and reflecting practical implementation outside a trial setting in a safety-net population. Follow-up was meaningful for a retrospective series with most patients demonstrating durable disease control, adding clinical context to the dosimetric and volumetric findings.

The main limitation of our study is the small sample size (*n* = 8), which limits statistical power, prevents robust subgroup analyses (including PD-L1 stratification), and increases sensitivity to individual patient-level events. We did not collect a concurrent, protocol-matched cohort treated without induction chemotherapy; the historical, sameinstitution comparisons discussed above are presented to contextualize toxicity, treatment duration, and brachytherapy-technique findings and cannot support causal inference about the incremental benefit of induction chemotherapy over high-quality brachytherapy alone. One patient was lost to follow up, which slightly constrains interpretation of durability and recurrence patterns, however, the median follow up was over 5 years.

It should be noted that all patients treated after August 2024 who received or were considered for induction chemotherapy had FIGO stage IVA-IVB disease which is more advanced than prior patients treated with induction at our institution. Following the publication of INTERLACE results in 2024, which showed 59% grade 3–4 adverse events during induction and 52% during CRT (following induction) compared to 48% during CRT for non-induction patients, there was a shift away from induction chemotherapy for non-stage IV disease (McCormack et al., 2024). Given the high rate of disease control via CRT followed by brachytherapy for less advanced disease, our clinicians generally began avoiding risking additional toxicity from induction chemotherapy at that time. However, for patients with more advanced disease, induction chemotherapy may be worth the risk of added toxicity to allow for brachytherapy eligibility and possible curative treatment.

### 4.3 Implications for practice and future research

This real-world series suggests that an INTERLACE-style induction chemotherapy approach may be a practical option for carefully selected patients with bulky, locally advanced cervical cancer, and that the resulting tumor regression may facilitate tandem-and-ovoid brachytherapy in patients who might otherwise require an interstitial approach. However, the frequency of hematologic toxicity, particularly during the chemoradiation phase, and the need for treatment modification in some patients emphasize the importance of patient selection, close monitoring, and supportive care. The ongoing NRG-GY037 trial and other prospective, multi-institutional or randomised comparisons will be important to determine whether induction chemotherapy provides incremental benefit beyond high-quality MRI-guided brachytherapy, including its effect on rectal dose feasibility and the need for interstitial technique. Future studies should also aim to identify predictors of patients who will benefit from this approach, including clinical factors and biomarkers such as PD-L1 status.

## 5 Conclusions

In this small, real-world cohort, induction chemotherapy followed by EMBRACE-style brachytherapy was feasible and associated with substantial tumor regression. HR-CTV doses aligned with contemporary targets and we observed manageable, phase-specific toxicity. Given the small sample size and absence of a concurrent comparison group, these findings are hypothesis-generating; larger, prospective, and comparative studies are needed to determine the incremental value of induction chemotherapy in this setting.

## CRediT authorship contribution statement

**Ashley Chavana:** Writing – review & editing, Writing – original draft, Software, Methodology, Formal analysis, Data curation, Conceptualization. **Madeline Flanagan:** Writing – review & editing, Data curation, Formal analysis, Investigation, Methodology. **Amir Owrangi:** Writing – review & editing, Validation, Supervision, Software, Methodology, Formal analysis, Data curation, Conceptualization. **Shelly Sharma:** Writing – review & editing, Supervision, Software, Methodology, Data curation. **Claire Hoppenot:** Writing – review & editing. **Jan Sunde:** Writing – review & editing. **Anthony B. Costales:** Writing – review & editing. **Spoorthi Kamepalli:** Writing – review & editing. **Tracilyn Hall:** Writing – review & editing. **Michelle Ludwig:** Writing – review & editing, Supervision, Software, Methodology, Investigation, Formal analysis, Data curation, Conceptualization.

## Ethics declaration

The authors have NOT obtained informed consent from participants or their legal representatives. Informed consent was waived due to the retrospective nature of the study.

This study was performed in compliance with relevant laws, regulatory frameworks and guidelines where the research took place. This study was approved by the Baylor College of Medicine. (Approval No. H- 47373).

## Declaration of competing interest

The authors declare that they have no known competing financial interests or personal relationships that could have appeared to influence the work reported in this paper.
